# Atherogenic Index of Plasma and Coronary Artery Disease in the Adult Population: A Meta-Analysis

**DOI:** 10.3389/fcvm.2021.817441

**Published:** 2021-12-16

**Authors:** Jing Wu, Qiang Zhou, Zhouxia Wei, Jinying Wei, Meizi Cui

**Affiliations:** ^1^Department of General Practice, The First Hospital of Jilin University, Changchun, China; ^2^Department of Cadre Ward, The First Hospital of Jilin University, Changchun, China

**Keywords:** atherogenic index of plasma, atherosclerosis, coronary artery disease, meta-analysis, inflammation-related atherosclerosis

## Abstract

**Background:** The atherogenic index of plasma (AIP), which is the logarithm of the ratio between the triglyceride and high-density lipoprotein cholesterol (TG/HDL-C) concentrations in molar units, is correlated with the burden of atherosclerosis. This study aimed to evaluate the association between the AIP and coronary artery disease (CAD) in the adult population by performing a meta-analysis.

**Methods:** Observational studies relevant for this meta-analysis were identified by searching the PubMed, Embase, and Web of Science databases. Only studies using multivariate analysis were considered. A random-effects model, which incorporates potential intra-study heterogeneity, was applied to combine the results.

**Results:** Ten observational studies were included. In studies with the AIP analyzed as a continuous variable, a higher AIP was associated with a higher odds of CAD (adjusted risk ratio [RR] per 1-standard deviation [SD] increment of AIP: 2.10, 95% confidence interval [CI]: 1.51–2.93, *P* < 0.001, I^2^ = 90%). Further analysis of studies with the AIP analyzed as a categorical variable showed a higher odds of CAD (adjusted RR: 2.35, 95% CI: 1.88–2.93, *P* < 0.001, I^2^ = 37%) in the participants with the highest versus the lowest AIP value. Subgroup analyses demonstrated consistent results in asymptomatic and symptomatic populations as well as in male and female participants (all between-group *P* values > 0.05).

**Discussion:** Current evidence, mostly from cross-sectional studies, suggests that a higher AIP value may be independently associated with CAD in the adult population.

## Introduction

Coronary artery disease (CAD) remains one of the major causes of morbidity and mortality in the global population (1–3). The basic pathological feature of CAD is systemic inflammation-related atherosclerosis (4). Clinically, an increased plasma low-density lipoprotein cholesterol (LDL-C) concentration has become a validated risk factor and a treatment target for CAD (5, 6). However, CAD remains in some patients who have achieved the recommended LDL-C level (7, 8). Subsequent studies have shown that the small dense low-density lipoprotein (sdLDL), a subfraction of LDL, may confer a more remarkable atherogenic efficacy than the overall LDL (9). Indeed, a recent meta-analysis including 21 studies has demonstrated that the amount of sdLDL is independently associated with the incidence of CAD (10). Moreover, sdLDL has been identified as a more atherogenic lipoprotein parameter than LDL-C and lipoprotein(a) in the Prospective Framingham Offspring Study (11). However, the methods for measuring sdLDL are technically complex and have a low cost-effectiveness, which limit its routine use in clinical practice (12). Previous studies have shown that the atherogenic index of plasma (AIP), which is the logarithm of the ratio between the triglyceride and high-density lipoprotein cholesterol (TG/HDL-C) concentrations in molar units, is well correlated to the diameter of LDL-C and reflective of the sdLDL level (13, 14). Although the AIP has been related to the severity of the atherosclerotic burden, inconsistent results have been observed in previous studies concerning the relationship between the AIP and CAD (15–24). In addition, the results of some studies have implied that a higher AIP value is correlated with CAD (15, 16, 18, 20–24), while others have not (17, 19). Moreover, it is still less known whether the potential association between the AIP and CAD is independent of the characteristics of the population. Therefore, this study aimed to evaluate the associations between the AIP and CAD in adults via a meta-analysis of previously published observational studies.

## Methods

The Meta-analysis of Observational Studies in Epidemiology (25) Statement and Cochrane's Handbook (26) were consulted during the design, execution, and reporting of this study.

### Literature Search

PubMed, Embase, and the Web of Science databases were searched using a combination of the following search terms: (1) “atherogenic index plasma” OR “AIP”; and (2) “atherosclerotic cardiovascular disease” OR “ASCVD” OR “cardiovascular events” OR “MACE” OR “cardiovascular” OR “coronary artery disease” OR “CAD” OR “CHD” on May 10, 2021. Only studies published in English were included. The references of related literature reports were also examined manually for possible available studies for the meta-analysis.

### Study Selection

The inclusion criteria were as follows: (1) design and form: observational studies in full-length articles; (2) participants: adults in a community or a hospital setting; (3) exposure: AIP; (4) outcome: CAD; and (5) data reported: the association between the AIP and CAD in multivariate analysis. The diagnosis of CAD was consistent with that applied in the original studies.

### Data Extraction and Quality Evaluation

Two authors performed the database search, literature review, data input, and study quality scoring independently, in accordance with the predefined criteria. If a disagreement occurred, it was resolved by consensus between the two authors. The following data were obtained for each study: (1) general information of the study; (2) characteristics of the study design; (3) participant information, such as the number of adults included, health status, and demographic features; (4) AIP presentation form (continuous or categorical); (5) definition of CAD; and (6) variables adjusted in the multivariate analyses. The Newcastle–Ottawa Scale (NOS) (27) was applied for study quality evaluation; the score ranges from 1 to 9 via three quality domains: participant selection, balance of characteristics between groups, and validation of outcome.

### Statistical Analyses

The risk ratio (RR) and the 95% confidence interval (CI) were used to indicate the association between the AIP and CAD. If the AIP was analyzed as a continuous variable in the original studies, the RR of CAD per 1-standard deviation (SD) increment of the AIP was used. If the AIP was analyzed as a categorical variable in the original studies, the RR of CAD in participants with the highest versus the lowest AIP level was extracted. Between-study heterogeneity was assessed by Cochrane's Q test and estimation of the I^2^ statistic (28). Significant heterogeneity was deemed if I^2^ > 50%. A random-effects model, which incorporates between-study heterogeneity, was used to pool the RR data (26). By omitting each study sequentially, sensitivity analyses were performed to evaluate the stability of the finding (29). Subgroup analyses were conducted to evaluate the impacts of the health status and the sex of the participants on the association. Potential publication bias was first assessed by symmetry inspection of the funnel plots and then by Egger's regression test (30). RevMan (Version 5.1; Cochrane Collaboration, Oxford, UK) software was used for the meta-analysis and statistical analysis.

## Results

### Literature Search

**Figure 1:**
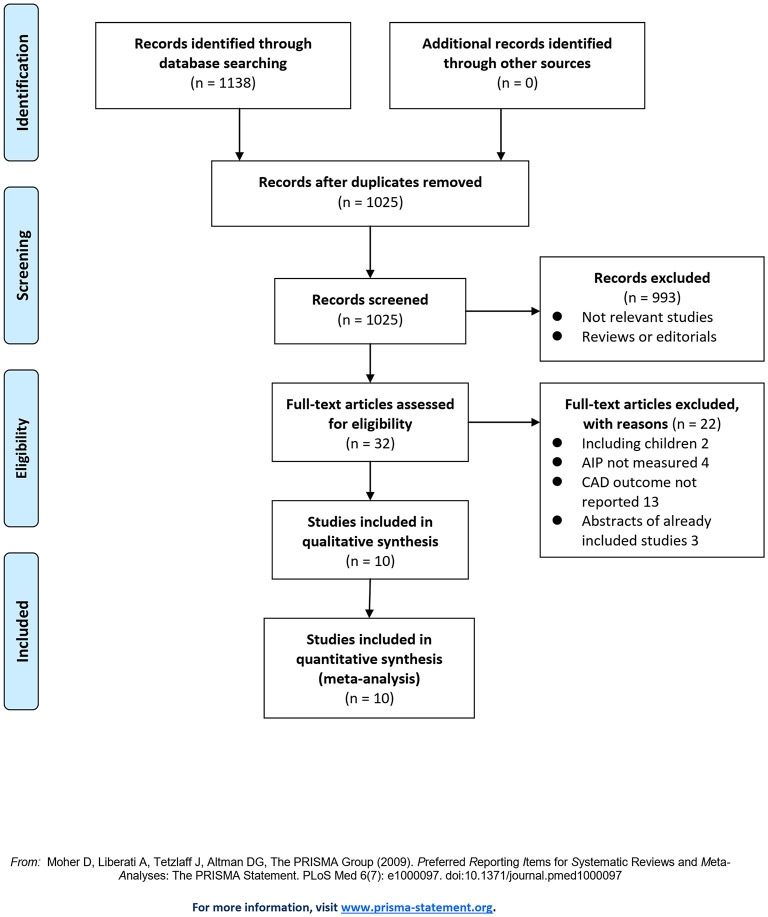
Flowchart of the database search and study identification.

The flowchart of the literature search and selection is shown in Figure 1. First, the literature search of PubMed, Embase, and the Web of Science databases retrieved 1,025 articles, of which 993 articles were not included based on irrelevant titles and abstracts. For the remaining 32 articles, full-text review was performed, and 22 articles were further judged to be unsuitable due to the reasons provided in Figure 1. Finally, ten studies were included in this meta-analysis (15–24).

### Study Characteristics and Quality Evaluation

Table 1 shows the general information of the included observational studies. Overall, one prospective cohort study (15), two case–control studies (16, 18), and seven cross-sectional studies (17, 19–24) comprising 29,847 adult participants were included. The studies were from Turkey (15), South Korea (21), and China (16–20, 22–24). Three of the studies included a community-derived population (15) or adults undergoing a health check-up (21, 22) who were asymptomatic, while the remaining seven studies included symptomatic participants who received coronary angiography (CAG) for the diagnosis of CAD (16–20, 23, 24). The number of participants in the study cohort varied from 463 to 6,928. The AIP was analyzed as a continuous variable in nine studies (15–21, 23, 24) and as a categorical variable in six studies (15, 17, 19, 21, 22, 24). The outcome of symptomatic CAD diagnosed by CAG was reported in eight studies (15–20, 23, 24), while subclinical CAD diagnosed by coronary computed tomography angiography was reported in two studies (21, 22). Conventional risk factors for CAD, such as age, sex, body mass index, hypertension, diabetes, and dyslipidemia, etc., were adjusted differently among the included studies. The NOS values were 6–8 for the observational studies, suggesting good quality.

**Table 1:** Characteristics of the included studies.

**References**	**Country**	**Design**	**Characteristics of participants**	**Number of participants**	**Mean age years**	**Male %**	**DM %**	**AIP analysis**	**Follow-up duration years**	**Outcome**	**Variables adjusted**	**Nos**.
Onat et al. (15)	Turkey	PC	Community- derived middle-aged adults	2,676	48.9	48.4	5.7	Continuous and categorical (Q4:Q1)	7.8	Symptomatic CAD	Age, sex, systolic BP, current smoking, BMI, and non-HDL cholesterol	8
Cai et al. (16)	China	CC	Adult patients undergoing CAG for suspected CAD	5,387	62.2	60.2	19.5	Continuous	NA	Symptomatic CAD diagnosed by CAG	Age, sex, smoking, DM, and hypertension	7
Ni et al. (17)	China	CS	Adult patients undergoing CAG for suspected CAD	463	65.3	66.1	17.9	Continuous and categorical (Q4:Q1)	NA	Symptomatic CAD diagnosed by CAG	Age, sex, BMI, FBG, Hcy, and smoking	7
Wu et al. (18)	China	CC	Postmenopausal women undergoing CAG for suspected CAD	696	61.7	0	22.2	Continuous	NA	Symptomatic CAD diagnosed by CAG	Age, hypertension, DM, smoking, heart rate, and FBG	7
Cai et al. (19)	China	CS	Adults ≤ 35 years old undergoing CAG for suspected CAD	1,478	33.0	92.9	9.0	Continuous and categorical (Q4:Q1)	NA	ACS diagnosed by CAG	Age, sex, smoking, hypertension, DM, and BMI	6
Won et al. (21)	South Korea	CS	Adults undergoing a health check-up	6,928	52.0	57.4	11.2	Continuous and categorical (Q4:Q1)	NA	Subclinical CAD diagnosed by CCTA	Age, sex, hypertension, DM, dyslipidemia, obesity, and proteinuria	7
Guo et al. (20)	China	CS	Postmenopausal women undergoing CAG for suspected CAD	4,644	64.2	0	30.6	Continuous	NA	Symptomatic CAD diagnosed by CAG	Age, BMI, smoking, alcohol drinking, hypertension, DM, dyslipidemia, and family history of CVD	8
Si et al. (22)	China	CS	Adults undergoing a health check-up	697	60.0	47.8	17.6	Categorized (median)	NA	Subclinical CAD diagnosed by CCTA	Age, sex, hypertension, DM, and dyslipidemia	7
Zhou et al. (24)	China	CS	Adult T2DM patients undergoing CAG for suspected CAD	3,278	59.0	75.4	100	Continuous and categorical (Q4:Q1)	NA	Symptomatic CAD diagnosed by CAG	Age, sex, BMI, systolic and diastolic BP, smoking, alcohol drinking, duration of T2DM, hypertension, dyslipidemia, and history of stroke	8
Wang et al. (23)	China	CS	Adult patients undergoing CAG for suspected CAD	3,600	60.4	62.3	24.2	Continuous	NA	Symptomatic CAD diagnosed by CAG	Age, sex, smoking, BMI, hypertension, DM, and dyslipidemia	8

*DM, diabetes mellitus; AIP, atherogenic index of plasma; NOS, Newcastle–Ottawa Scale; PC, prospective cohort; CC, case-control; CS, cross-sectional; CAD, coronary artery disease; CAG, coronary angiography; T2DM, type 2 diabetes mellitus; Q, quartile; NA, not applied; CCTA, coronary computed tomography angiography; BP, blood pressure; BMI, body mass index; HDL, high-density lipoprotein; CVD, cardiovascular disease*.

### Association Between the AIP and CAD

**Figure 2:**
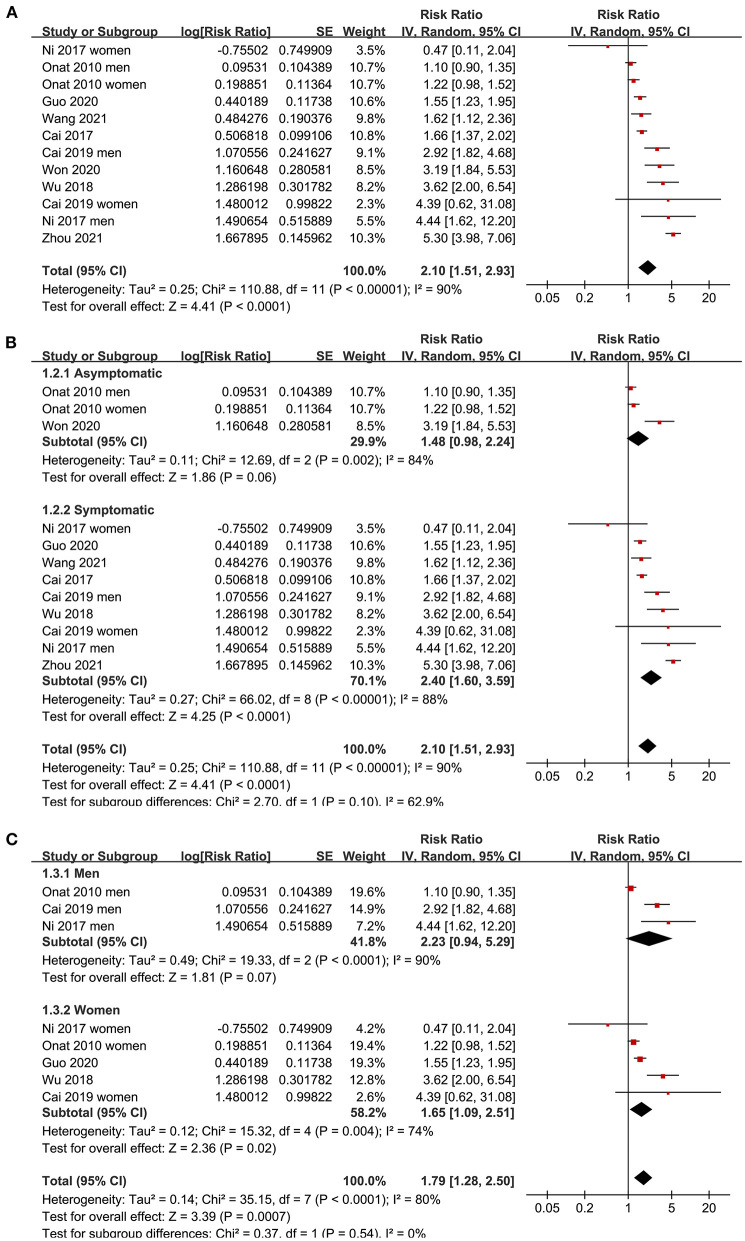
Forest plots for the meta-analysis of the association between the AIP analyzed as a continuous variable and the odds of CAD in the adult population. **(A)** Overall meta-analysis; **(B)** Subgroup analysis according to the health status of the participants; and **(C)** Subgroup analysis according to the sex of the participants.

**Figure 3:**
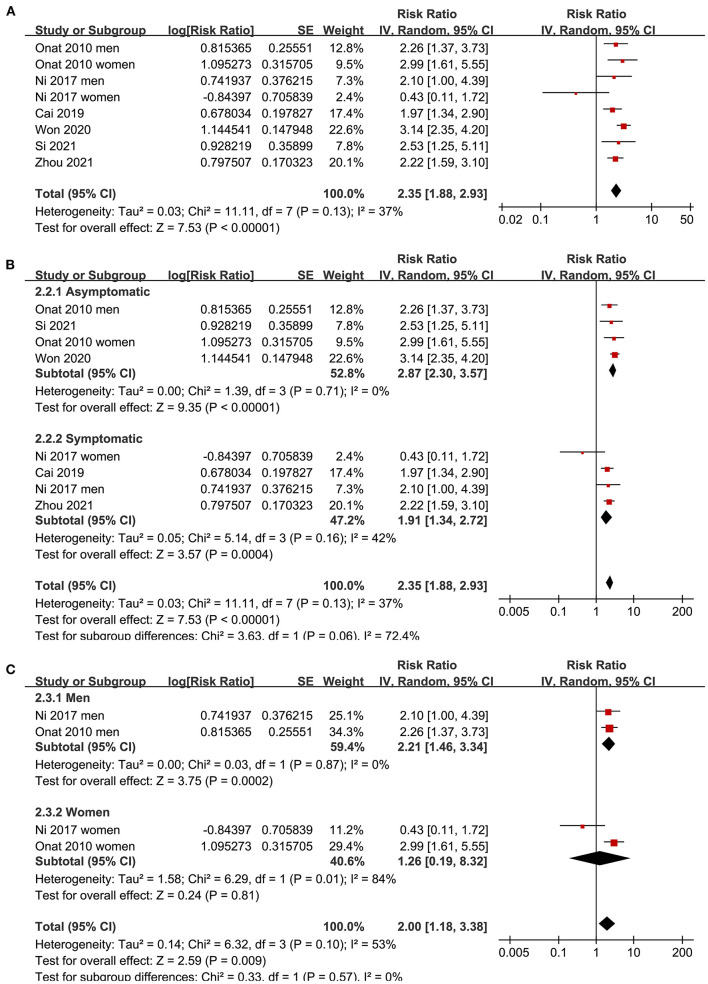
Forest plots for the meta-analysis of the association between the AIP analyzed as a categorical variable and the odds of CAD in the adult population. **(A)** Overall meta-analysis; **(B)** Subgroup analysis according to the health status of the participants; and **(C)** Subgroup analysis according to the sex of the participants.

Since three studies (15, 17, 19) reported data in men and women separately, these datasets were independently included in the meta-analysis. The pooled results of 12 datasets from nine studies (15–21, 23, 24) with the AIP analyzed as a continuous variable showed that a higher AIP was associated with a higher odds of CAD (adjusted RR for 1-SD increment of AIP: 2.10, 95% CI: 1.51–2.93, *P* < 0.001; Figure 2A) with significant heterogeneity (P for Cochrane's Q test < 0.01, I^2^ = 90%). Sensitivity analysis by excluding one dataset at a time showed consistent results (RR: 1.8–2.22, all *P* < 0.05). In addition, subgroup analyses showed consistent results in asymptomatic and symptomatic populations as well as in male and female participants (all P for subgroup analyses > 0.05; Figures 2B,C). Further analysis including eight datasets from six studies (15, 17, 19, 21, 22, 24) with the AIP analyzed as a categorical variable showed that compared to those with the lowest AIP level, the participants with the highest AIP level were also associated with a higher odds of CAD (adjusted RR: 2.35, 95% CI: 1.88–2.93, *P* < 0.001; Figure 3A) with moderate heterogeneity (P for Cochrane's Q test = 0.13, I^2^ = 37%). Sensitivity analysis by omitting one dataset at a time did not significantly affect the results (RR: 2.16–2.42, all *P* < 0.05). Furthermore, subgroup analyses did not show that the health status or the sex of the participants significantly influenced the association between the AIP and CAD (all *P* for subgroup analyses > 0.05; Figures 3B,C).

### Publication Bias

**Figure 4:**
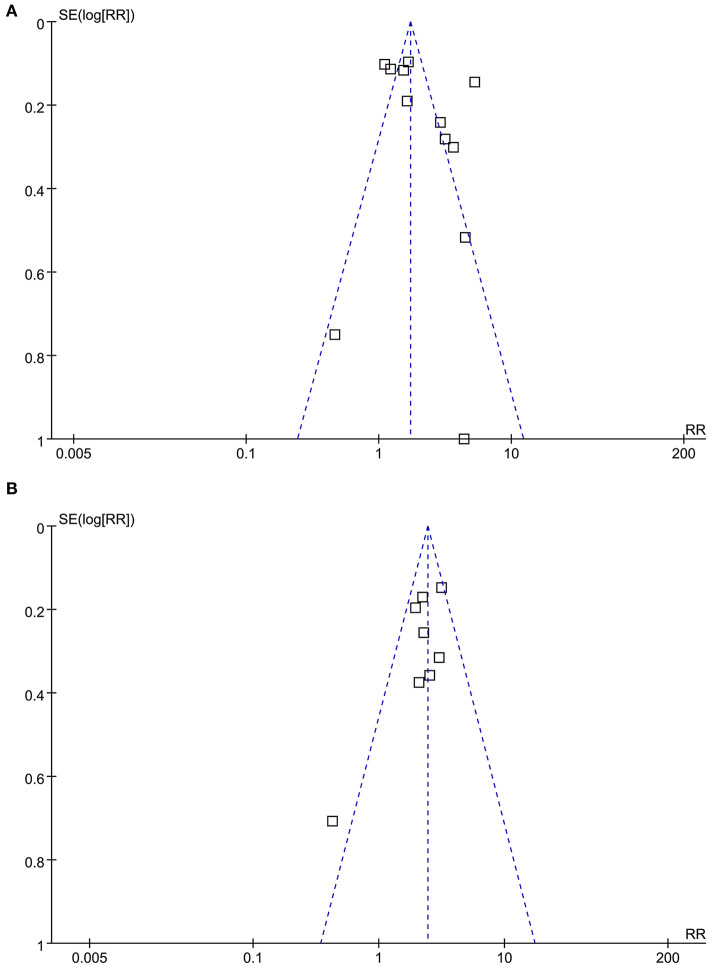
Funnel plots for the publication bias underlying the meta-analysis of the association between the AIP and CAD. **(A)** Funnel plots for the meta-analysis of the AIP analyzed as a continuous variable; and **(B)** Funnel plots for the meta-analysis of the AIP analyzed as a categorical variable.

As shown in Figure 4, the funnel plots for the association between the AIP and CAD were symmetric, suggesting no significant publication bias. The results of Egger's regression tests also did not support significant publication bias for the meta-analyses (*P* = 0.285 and 0.311, respectively).

## Discussion

In this meta-analysis of observational studies, we found that a higher AIP was independently correlated with higher odds of CAD after adjusting for conventional cardiovascular risk factors. The results with AIP as a continuous variable compared to as a categorical variable were consistent. Further sensitivity analyses by excluding one study at a time did not significantly affect the results, indicating the robustness of the findings. In addition, the results of predefined subgroup analysis showed that the association between the AIP and CAD was not significantly affected by the health status or the sex of the patients. Taken together, these results support that the AIP may be an independent risk factor for CAD in the adult population.

To the best of our knowledge, this may be the first meta-analysis that summarizes the current knowledge regarding the association between the AIP and CAD in the adult population. Several methodological strengths of this study should be noted before the clinical implications of the findings are discussed. First, an extensive search strategy was applied, aiming to incorporate all eligible studies. In addition, studies with the AIP analyzed as a continuous variable and as a categorical variable were pooled separately, and the consistent results further indicated the validity of the findings. Moreover, only studies with multivariate analysis were included, thus providing a potentially independent association between the AIP and CAD. Finally, multiple sensitivity and subgroup analyses further indicated the stability of the findings. The results of the meta-analysis were consistent with previous studies showing a close correlation between the AIP and cardiovascular risks. For instance, a cross-sectional study including 340 healthy women has revealed that the AIP level is significantly correlated with the Framingham risk score (FRS), indicating the possible role of the AIP in the early diagnosis of CAD (31). The association between the AIP and cardiovascular risks is also evident in young adults. A recent study including people aged 18–22 years old has demonstrated that a higher AIP value is associated with multiple risk factors for CAD, such as hyperlipidemia, hypertension, hyperuricemia, and metabolic syndrome (32). The association between a higher AIP value and an increased risk of CAD as indicated by an increased FRS was even consistent in patients with some specific medical situations, such as those with HIV infection (33) and women with rheumatoid arthritis or systemic lupus erythematosus (34). The potential independent association between the AIP and CAD even after adjusting for multiple conventional CAD risk factors may be explained by the fact that the AIP value could more comprehensively reflect the extent of metabolic disorder than a single parameter such as LDL-C. As mentioned previously, the AIP could indirectly reflect the level of sdLDL, which is a subfraction of LDL-C with a stronger atherogenic effect (35, 36). Moreover, a higher AIP value has been associated with multiple other risk factors that are involved in the pathogenesis of CAD, such as hyperuricemia (37), abdominal obesity (38), declining renal function (39), and lack of physical activity (40). Since the AIP can be easily obtained via routine analysis of blood lipid levels, the results of our meta-analysis support the incorporation of the AIP into the risk stratification for CAD.

Our study also has some limitations that must be addressed. First, only one of the included studies was a cohort study. A sequential relationship between a higher AIP value and an increased risk of CAD should be further validated in large-scale prospective studies. Second, the meta-analysis was based on data at the study level rather than for individual patients. We could not determine whether a difference in participant characteristics, such as age, ethnicity, and comorbidities, could be a source of heterogeneity among the included studies. In addition, although only studies with multivariate analysis were included, there might be residual factors that may confound the association between the AIP and CAD, such as dietary factors and concurrent medications that may affect TG and/or HDL-C levels. Finally, an optimal cutoff value of the AIP for the prediction of CAD risk remains to be determined, and it continues to be unknown whether incorporation of the AIP into the current risk stratification process for CAD, such as the FRS, is associated with an improved predictive efficacy. Therefore, future studies are necessary.

## Conclusions

In conclusion, the results of this meta-analysis suggest that a higher AIP value may be independently associated with a higher odds of CAD in the adult population. Large-scale prospective cohort studies are warranted to validate these findings and to evaluate the possible role of the AIP as a predictor for CAD risk in the general adult population.

## Data Availability Statement

The original contributions presented in the study are included in the article/Supplementary Material, further inquiries can be directed to the corresponding author.

## Author Contributions

JWu, QZ, ZW, and MC conceived and designed research. JWu, QZ, and JWe collected data and conducted research. JWu, ZW, and MC analyzed and interpreted data. JWu and QZ wrote the initial paper. MC and QZ revised the paper. MC had primary responsibility for final content. All authors read and approved the final manuscript.

## Funding

This work was supported by China National Natural Science Foundation (81702597) and Research Fund of the First Hospital of Jilin University (04020940001 and JDYY11202036).

## Conflict of Interest

The authors declare that the research was conducted in the absence of any commercial or financial relationships that could be construed as a potential conflict of interest.

## Publisher's Note

All claims expressed in this article are solely those of the authors and do not necessarily represent those of their affiliated organizations, or those of the publisher, the editors and the reviewers. Any product that may be evaluated in this article, or claim that may be made by its manufacturer, is not guaranteed or endorsed by the publisher.
