# Corrigendum: Atherogenic index of plasma and coronary artery disease in the adult population: A meta-analysis

**DOI:** 10.3389/fcvm.2023.1153914

**Published:** 2023-03-08

**Authors:** Jing Wu, Qiang Zhou, Zhouxia Wei, Jinying Wei, Meizi Cui

**Affiliations:** ^1^ Department of General Practice, the First Hospital of Jilin University, Changchun, China; ^2^ Department of Cadre Ward, the First Hospital of Jilin University, Changchun, China

**Keywords:** atherogenic index of plasma, atherosclerosis, coronary artery disease, inflammation-related atherosclerosis, meta-analysis

Corrigendum on Atherogenic index of plasma and coronary artery disease in the adult population: A meta-analysis By Wu J, Zhou Q, Wei Z, Wei J and Cui M. (2021) Front. Cardiovasc. Med. 8:817441. doi: 10.3389/fcvm.2021.817441

**Error in Author List**

In the published article, there was an error in the author list which did not clarify the equal contributions of authors Jing Wu and Qiang Zhou. The corrected author list appears below.

Jing Wu^1†^, Qiang Zhou^2†^, Zhouxia Wei^2^, Jinying Wei^1^, Meizi Cui^2*^

†These authors contributed equally to this work and share first authorship

The authors apologize for this error and state that this does not change the scientific conclusions of the article in any way. The original article has been updated.

