# Supplementary material for: Atherogenic Index of Plasma and Coronary Artery Disease in the Adult Population: A Meta-Analysis
Source: Front Cardiovasc Med. 2021 Dec 16;8:817441. doi: 10.3389/fcvm.2021.817441 (PMC8716758; doi:10.3389/fcvm.2021.817441)
Supplement: Supplementary file 1 [file Data_Sheet_1.docx]

**Identification of studies via databases and registers**

Records removed *before screening*:

Duplicate records removed (n = 113)

Records identified from*:

Databases (n = 1138)

**Identification**

Records screened

(n = 1025)

Records excluded**

(n = 993)

Not relevant stuides or reviews

Reports sought for retrieval

(n = 32)

Reports not retrieved

(n = 0)

**Screening**

Reports assessed for eligibility

(n = 32)

Reports excluded:

Including children (n = 2)

AIP not measured (n = 4)

CAD outcome not reported (n = 13)

Abstracts of already included studies (n = 3)

Studies included in review

(n = 10)

Reports of included studies

(n = 10)

**Included**

*Consider, if feasible to do so, reporting the number of records identified from each database or register searched (rather than the total number across all databases/registers).

**If automation tools were used, indicate how many records were excluded by a human and how many were excluded by automation tools.

*From:*  Page MJ, McKenzie JE, Bossuyt PM, Boutron I, Hoffmann TC, Mulrow CD, et al. The PRISMA 2020 statement: an updated guideline for reporting systematic reviews. BMJ 2021;372:n71. doi: 10.1136/bmj.n71

For more information, visit: <http://www.prisma-statement.org/>
